# Incompatible Medicines

**Published:** 1880-07

**Authors:** 


Incompatible Medicines.—One of the most important steps towards true science in the administration and use of medicines, has been made in the discovery within the past few years of their physiological action on the animal economy in health and the antagonism, or absolute incompatibility of certain drugs with other, when taken into the system. Should the examinations and verifications, which have yielded such valuable results heretofore, be continued in the spirit that has characterized them, for the next decade, a degree of positiveness and *certainty* never known before must distinguish the prescriptions of the more intelligent practitioners of medicine, and then will probability and verisimilitude be relegated to the domain of the past, or compelled to take up their abode with the quacks and juggles of the art. The direct antagonism between veratrum veride and opium for example, is known and recognized by every one who strives to practice the profession of medicine as far as possible on scientific principles! And yet we learn that a certain "professor" of practical medicine, in recommending the use of the first named article in *peneumonia* to *his* class, actually instructed them to give *five drops each* of this and laudanum, every two or three hours. Shades of Norwood! What of your teaching and experience? And thus this "blind guide," goes on advising five drops each of tincture veratrum and laudanum, every two or three hours!

